# Development and Characterization of Anti-*Naja ashei* Three-Finger Toxins (3FTxs)-Specific Monoclonal Antibodies and Evaluation of Their In Vitro Inhibition Activity

**DOI:** 10.3390/toxins14040285

**Published:** 2022-04-16

**Authors:** Ernest Z. Manson, Mutinda C. Kyama, Josephine Kimani, Aleksandra Bocian, Konrad K. Hus, Vladimír Petrilla, Jaroslav Legáth, James H. Kimotho

**Affiliations:** 1 Institute for Basic Sciences, Technology & Innovation, Pan African University, Nairobi 00100, Kenya; 2 Department of Medical Laboratory Science, College of Health Sciences, Jomo Kenyatta University of Agriculture & Technology, Nairobi 00100, Kenya; mkyama@jkuat.ac.ke; 3 Department of Biochemistry, College of Health Sciences, Jomo Kenyatta University of Agriculture & Technology, Nairobi 00100, Kenya; jkimani@jkuat.ac.ke; 4 Department of Biotechnology and Bioinformatics, Faculty of Chemistry, Rzeszow University of Technology, 35-959 Rzeszow, Poland; bocian@prz.edu.pl (A.B.); k.hus@prz.edu.pl (K.K.H.); jaroslav.legath@uvlf.sk (J.L.); 5 Department of Biology and Physiology, University of Veterinary Medicine and Pharmacy, 041-81 Košice, Slovakia; vladimir.petrilla@uvlf.sk; 6 Zoological Department, Zoological Garden Košice, Široká 31, 040-06 Košice-Kavečany, Slovakia; 7 Department of Pharmacology and Toxicology, University of Veterinary Medicine and Pharmacy, 041-81 Košice, Slovakia; 8 Kenya Medical Research Institute, Nairobi 00100, Kenya; jhkimotho@kemri.org

**Keywords:** *Naja ashei*, three-finger toxins, monoclonal antibodies, snakebite envenoming, inhibition ELISA

## Abstract

Antivenom immunotherapy is the mainstay of treatment for snakebite envenoming. Most parts of the world affected by snakebite envenoming depend on broad-spectrum polyspecific antivenoms that are known to contain a low content of case-specific efficacious immunoglobulins. Thus, advances in toxin-specific antibodies production hold much promise in future therapeutic strategies of snakebite envenoming. We report anti-3FTxs monoclonal antibodies developed against *N. ashei* venom in mice. All the three test mAbs (P4G6a, P6D9a, and P6D9b) were found to be IgG antibodies, isotyped as IgG1. SDS-PAGE analysis of the test mAbs showed two major bands at approximately 55 and 29 kDa, suggestive of immunoglobulin heavy and light chain composition, respectively. The immunoaffinity-purified test mAbs demonstrated higher binding efficacy to the target antigen compared to negative control. Similarly, a cocktail of the test mAbs was found to induce a significantly higher inhibition (*p*-value < 0.0001) compared to two leading commercial brands of antivenoms on the Kenyan market, implying a higher specificity for the target antigen. Both the test mAbs and 3FTxs polyclonal antibodies induced comparable inhibition (*p*-value = 0.9029). The inhibition induced by the 3FTxs polyclonal antibodies was significantly different from the two antivenoms (*p*-value < 0.0001). Our results demonstrate the prospects of developing toxin-specific monoclonal-based antivenoms for snakebite immunotherapy.

## 1. Introduction

Snakebite envenoming has assumed a public health dimension at the global level [1]. It is considered one of the most discussed medical issues in the world due to the constant human–snake interactions, especially in the tropical and sub-tropical regions [2,3]. Of the close to 5.4 million people who are bitten by snakes each year globally [4], an estimated 81,000–138,000 deaths and 400,000 cases of permanent disabilities and disfigurements are involved [1]. In Africa, where data on snakebites and snakebite envenoming are limited, nearly 1 million snakebites occur every year [5], resulting in about 32,000 deaths [6] and 8000 amputations [7]. Although believed to be underreported, an estimated 15,000 snakebites occur in Kenya yearly. Of the 29 venomous snake species in Kenya, 13 are known to be medically significant, with bites resulting from only nine believed to require medical attention [8]. *Naja ashei*, commonly known as the large brown spitting cobra, is among the snakes that pose a significant threat to public health [9] and thus is categorized as a medically important species not only in Kenya, but also Tanzania, Uganda, Somalia, and Ethiopia [10].

The venom of *N. ashei* comprises of complex toxins and proteins including three-finger toxins (3FTxs), phospholipase A_2_ (PLA_2s_), snake venom 5′-nucleotidases (5′N), cobra venom factor (CVF), venom nerve growth factor (VNGF), and snake venom metalloproteinases (SVMPs). Consistent with other African spitting cobra snakes, *N. ashei* venom is understood to provoke cytotoxic effects; hence, tissue necrosis which is attributable to the preponderance of three-finger toxins (69%) and phospholipase A_2_ proteins (27%) [11,12,13,14]. Three-finger toxins are either neurotoxic or cytotoxic [15].

Antivenom immunotherapy remains the most effective and the only valid treatment option for snakebite envenoming. The neutralizing efficacy of conventional antivenoms of venom-induced lethality and other toxic effects has improved owing to, among other things, advancements in protein chemistry and immunology, introduction of new adjuvants and enhanced antibody isolation and characterization assays. In spite of these advancements, the risks of adverse effects persist [16,17]. Antivenoms that are rich in antibody-binding fragments (Fab’_2_) are believed to be as effective as intact IgGs at neutralizing venom toxins. Nevertheless, their associated adverse effects and potential to activate host complement system linger [18,19].

To prepare conventional antivenoms, horses and in some cases sheep are immunized with venom from a single snake or a pool of venoms from several species. The process of immunizing these animals is intended to elicit an immune response, culminating in high antibody levels that are able to recognize, bind, and abrogate key venom toxins. However, the process also generates undesirable antibodies that are targeted at non-toxic venom components and irrelevant venom epitopes. Thus, it is argued that close to 95% of IgGs that constitute current antivenoms do not have therapeutic value [20]. These irrelevant proteins are thought to play a role in the overall antivenom therapy side effects [20]. Conventional antivenoms in their current state are limited in their efficacy to neutralize certain effects of envenoming including localized tissue damage. Additionally, only an average of 12.5% of IgGs per vial of antivenom is believed to be specific against venom toxins [1]. Furthermore, where venoms of phylogenetically diverse snakes are used to immunize an animal, a greater number of IgG specificities are generated in the animal, suggesting that the proportion of total IgGs that are targeted at any one snake is small, a situation which warrants more vials in order to achieve clinical cure. Ultimately, this gives rise to a therapeutic paradox, given that any additional vial of antivenom increases not only the risk of antivenom-induced side effects but also the cost of treatment [1]. Therefore, cocktails of monoclonal antibodies targeted at a range of epitopes may offer advantages in terms of higher specificity and the use of fewer antivenom vials, and ultimately the achievement of desirable immunotherapy [21]. Toxin-specific antibodies not only lead to an increase in the dose-efficacy of antivenoms but also reduce the risk of anaphylactic and late serum reactions that characterize the administration of large volumes of horse and sheep-derived antivenoms [22]. The aim of the current work was to develop three-finger toxins-specific monoclonal antibodies in mice and analyze their in vitro activity in inhibiting 3FTxs proteins present in crude *N. ashei* venom.

## 2. Results

### 2.1. Immune Response against 3FTxs Antigen and Antibody Titer Determination

**Figure 1:**
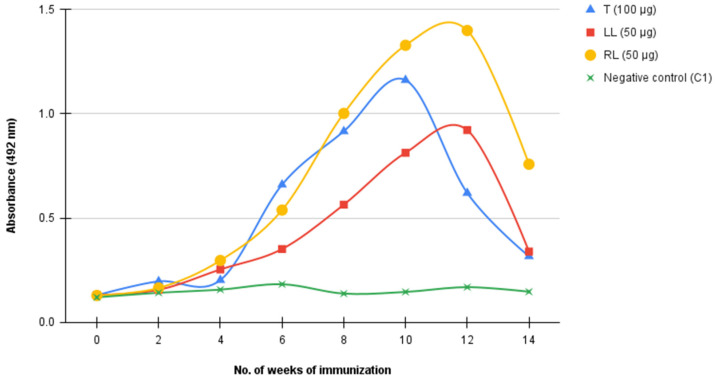
Immune response of Balb/c mice immunized with three-finger toxin antigen. Toxin was attenuated in 0.125% glutaradehyde, mixed with adjuvant, and administered intraperitoneally. Mice were bled in the tail vein for antibody titer determination and boosters administered every two weeks. T: mouse immunized with 50 µg inactivated antigen; LL: mouse immunized with 50 µg inactivated antigen; RL: mouse immunized with 100 µg inactivated antigen; and negative control: mouse immunized with PBS.

Pre-immune, toxin-challenged, and PBS-only immunized mice serum samples were assayed to determine antibody titers. It was found that various levels of immune response were elicited against the toxin. Although two mice each received 50 µg of inactivated antigen, varied immune responses were obtained, with one mouse producing a higher titre (OD_492_ = 1.400) and the other a lower titre (OD_492_ = 0.922) than the mouse immunized with 100 µg (OD_492_ = 1.162). Antibody production generally peaked between 10–12 weeks. No immune response was observed in the PBS-only immunized control. Immune response against the toxin was also observed to drop after 12 weeks of immunization (Figure 1).

### 2.2. Characterization of mAbs

**Figure 2:**
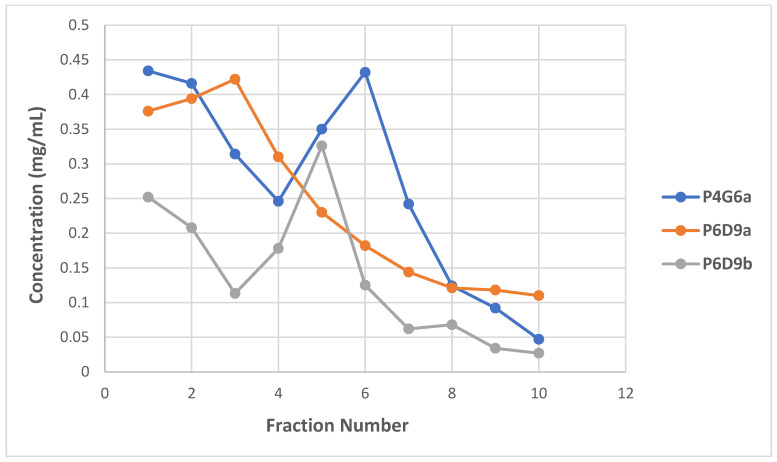
Determination of immunoglobulin (IgG) yield of hybridoma clones. Antibodies were eluted in 1 mL fractions (a total of 10) per clone and immunoglobulin yield determined using the Qubit Protein Assay Kit.

As demonstrated by representative clones, P4G6a and P6D9b antibody concentration peaked at fractions six and five, respectively. Similarly, the concentration of P6D9a peaked at fraction three. The concentrations of the clones were found to be 0.432, 0.422, and 0.326 mg/mL for P4G6a, P6D9a, and P6D9b, respectively. Thus, for each clone, three best performing fractions were selected for downstream applications (Figure 2). The generated hybridomas were found to be IgG-secreting clones. All three mAbs were isotyped as IgG1.

#### SDS-PAGE Analysis of mAbs

**Figure 3:**
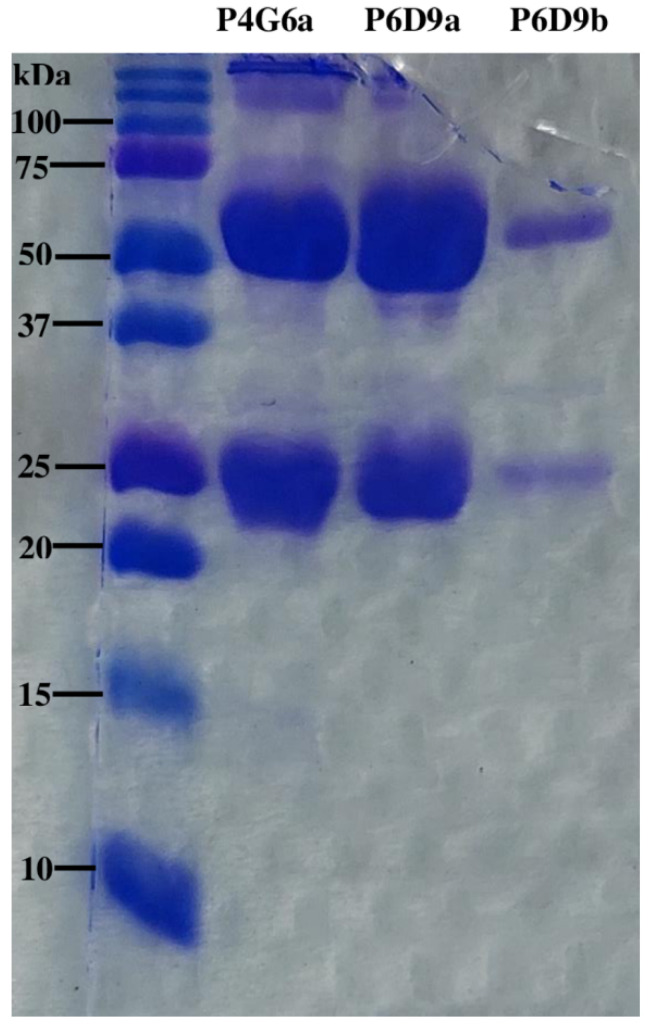
SDS-PAGE analysis of immunoaffinity-purified mAbs. Left lane—molecular weight marker (10–250 kDa); lane 1—clone P4G6a mAb; lane 2—clone P6D9a mAb; and lane 3—clone P6D9b mAb.

All three mAbs were analyzed immunochemically to determine their purity. When subjected to SDS-PAGE analysis, P4G6a, P6D9a, and P6D9b all showed two major protein bands at approximately 55 and 29 kDa (Figure 3).

### 2.3. Evaluating Binding Efficacy of Purified mAbs

**Figure 4:**
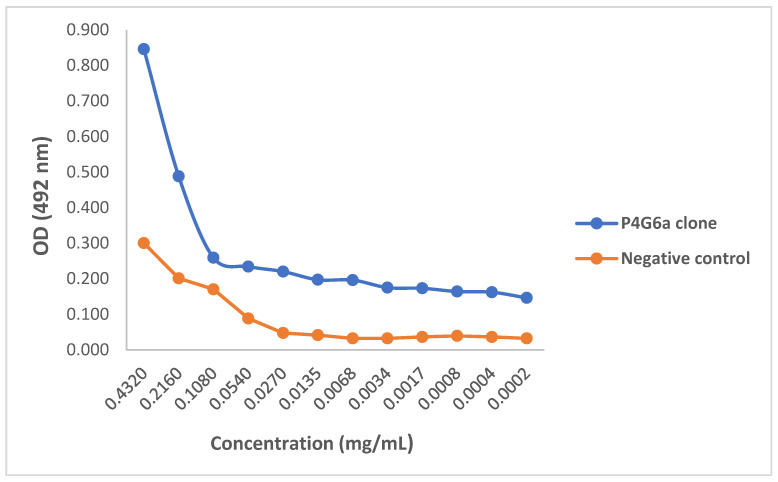
ELISA titration of monoclonal antibody P4G6a. ELISA plate was coated with 1 µg/mL purified 3FTx antigen and incubated with serially diluted P4G6a monoclonal antibody (0.4320 to 0.0002). Pre-immune serum was used as negative control.

**Figure 5:**
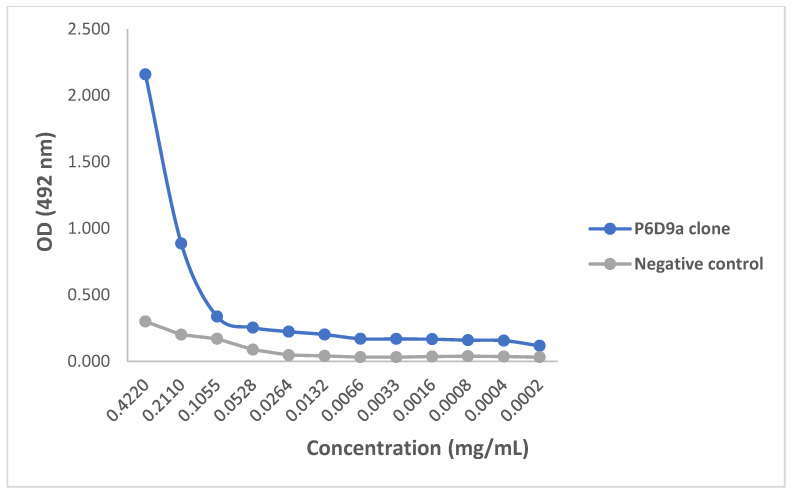
ELISA titration of monoclonal antibody P6D9a. ELISA plate was coated with 1 µg/mL purified 3FTx antigen and incubated with serially diluted P6D9a monoclonal antibody (0.4220 to 0.0002). Pre-immune serum was used as negative control.

**Figure 6:**
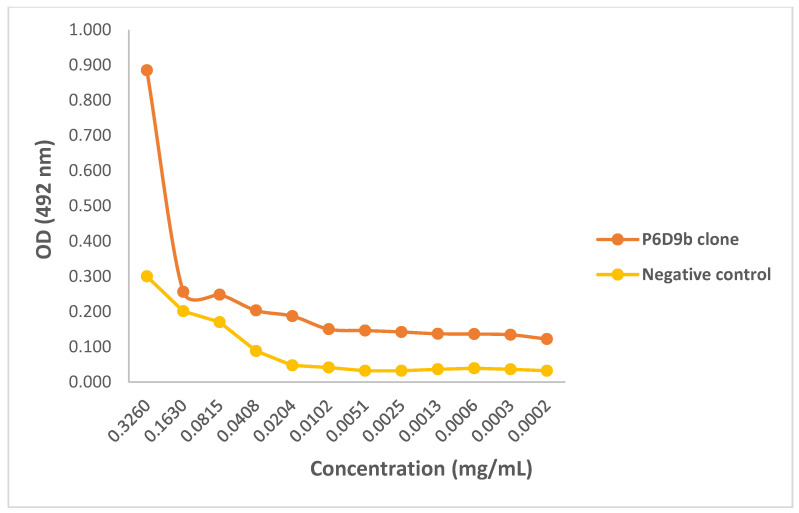
ELISA titration of monoclonal antibody P6D9b. ELISA plate was coated with 1 µg/mL purified 3FTx antigen and incubated with serially diluted P6D9b monoclonal antibody (0.3260 to 0.0002). Pre-immune serum was used as negative control.

Preliminary activity of the purified mAbs against 3FTx antigen was assessed by testing the ability of the mAbs to identify and bind to the target antigen in an ELISA titration. All three mAbs demonstrated capacity to recognize 3FTxs, and thus were able to bind to the antigen at the lowest concentration tested (0.0002 mg/mL). When compared with the control sample, all three clones were found to bind to the target antigen with comparatively higher efficacy as assessed by optical density. Again, for all three mAbs, there was activity at higher concentrations following which the activity dropped and flattened (Figure 4, Figure 5 and Figure 6).

### 2.4. Evaluating In Vitro Activity of Purified mAbs

**Figure 7:**
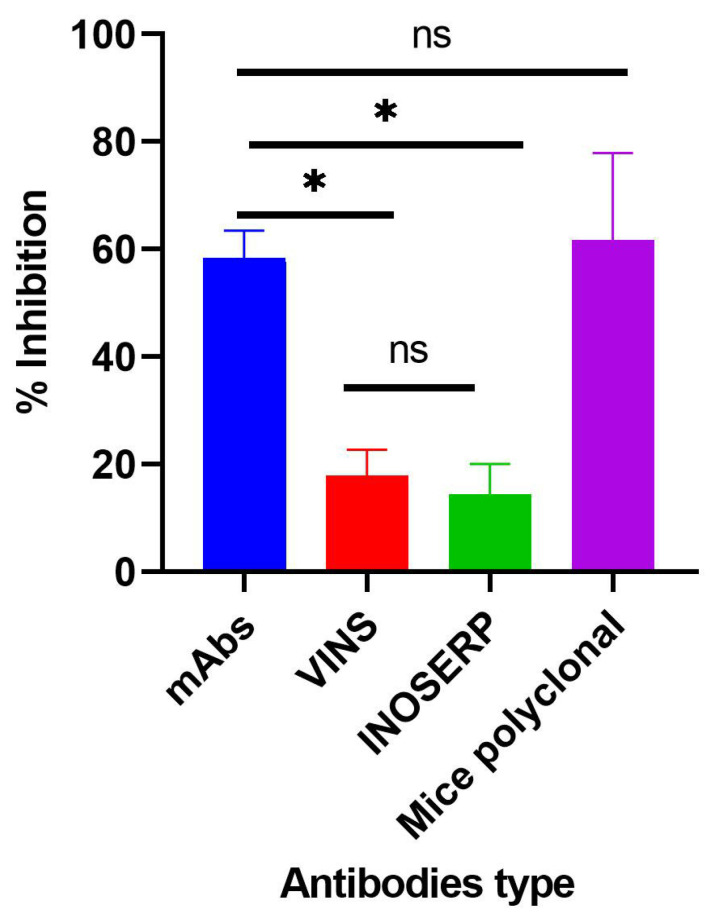
Comparison of the inhibitory effect of a cocktail of purified test mAbs, selected commercial antivenoms, and mice polyclonal antibodies. Purified mAbs, VINS^TM^, and Inoserp^TM^ antivenoms and polyclonal antibodies were diluted two-fold and assayed. Sample antigen was serially diluted three-fold from 27 µg/mL to 0.04 µg/mL. * siginificant difference; ns: difference not statistically significant.

Further confirmation of the recognition, binding, and activity of the mAbs was evaluated using an inhibition ELISA assay. A cocktail of the purified mAbs was tested against two commercial antivenoms available in the Kenyan market (VINS^TM^ and Inoserp^TM^) and 3FTxs-challenged mice polyclonal antibodies. Tukey’s multiple comparison test showed that the 3FTxs-induced inhibition by the test mAbs was significantly different when compared with inhibition by the two antivenoms (*p*-value < 0.0001). Both the mAbs and polyclonal antibodies induced comparable inhibition (*p*-value = 0.9029). Similarly, there was a significant difference in the inhibition induced by the polyclonal antibodies relative to both antivenoms (*p*-value < 0.0001) (Figure 7).

## 3. Discussion

The current study was aimed at developing anti-3FTxs monoclonal antibodies and subsequently evaluating their activities in vitro. Three mAbs were successfully developed and the activity of these mAbs tested against two commercial antivenoms and 3FTxs-challenged polyclonal antibodies. Highly toxic venom components, especially 3FTxs, are known to be poorly immunogenic [23,24]. In spite of this, it was possible to elicit immune response culminating in the production of antibodies against the toxin in all three immunized mice. This observation is similar to that reported by Laustsen and colleagues, in which 3FTxs and PLA_2_s-immunized mice provoked varied immune responses, although neither toxin was found to be highly immunogenic. PLA_2_s-challenged mice were found to elicit a slightly higher immune response relative to 3FTxs, a finding attributable to the smaller molecular weight of the latter [25]. This position is supported by Bermúdez-Méndez and colleagues who observed that the immune response to a toxin might be functionally related to its molecular weight [13]. The observed drop in antibody titers between week 12 and 14 may be due to the cumulative effect of the attenuating agent on the toxin. Regardless of the method of attenuation, Moroz-Perlmutter and colleagues contended that these treatments often result in not only a destruction of the toxic sites but also the immunogenic sites of the toxin [26]. As a consequence, this may alter or interfere with the normal immune response against immunized antigens.

The hybridomas resulting from the fusion of SP2/0 myeloma cells and mouse spleen cells demonstrated that IgG antibodies were the dominant class produced as confirmed by the isotyping test. Observations made by Williams and colleagues found that second-generation antivenoms which are affinity chromatography-purified contain only intact IgGs and are known to decrease the risk of antivenom-induced adverse effects [15]. Cocktails of mAbs that target various antigenic epitopes are known to offer advantages in terms of higher specificity, requirement for fewer vials, and minimal adverse effects [15,22,27]. Hence, these mouse mAbs could potentially reduce the adverse effects that typify the administration of large volumes of horse-derived antivenoms. Additionally, the determination of the isotypes may be relevant in selecting a suitable proteolytic method for the production of antibody fragments. The presence of two major bands at approximately 55 and 29 kDa through SDS-PAGE analysis were indicative of the heavy and light chain composition of immunoglobulins, respectively [21]. This finding appears to further confirm the monoclonality of the antibodies generated and underscores the result of the isotyping test.

A concentration-dependent reduction in binding of the test mAbs was observed. The ODs and binding patterns observed may suggest that the effectiveness of a mAb to bind to its target antigen may, to a large extent, be dictated by its concentration as demonstrated by the titration assay. The diversity in the detection of the 3FTx antigen by the different mAbs is supported by Leow and colleagues who reported variations in sensitivity between two mAbs in detecting recombinant malaria proteins [28]. Additionally, the ability of test mAbs to recognize snake venom-derived target antigens via ELISA has been demonstrated in previous studies [21,29]. In confirming the recognition, binding, and further activity of the mAbs relative to commonly used commercial antivenoms, a cocktail of the purified mAbs comparatively induced significantly higher inhibition than both antivenoms but was comparable to that induced by the polyclonal antibodies. This can be attributed to the higher specificity of the *N. ashei* anti-3FTxs test monoclonal antibodies and 3FTxs polyclonal antibodies to the target antigen. Although both commercial antivenoms contained anti-3FTx antibodies, these antibodies were raised against other *Naja* snakes since neither antivenom contains *N. ashei* venom as part of the immunizing mixtures [10]. Thus, the findings underscore the specificity of toxin-specific antibodies and to some extent, a known limitation in polyvalent anti-snake antibodies, particularly when venoms from phylogenetically diverse snakes are used for immunization.

## 4. Conclusions

Our study reports for the first time *N. ashei* anti-3FTxs murine monoclonal antibodies. The results show that the individual mAbs were able to bind to the target antigen at a relatively higher efficacy as assessed by optical density compared to the negative control (P4G6a: OD_492_ = 0.846; P6D6a: OD_492_ = 2.159; P6D6b: OD_492_ = 0.885; negative control: OD_492_ = 0.300). When combined as a cocktail, the mAbs induced significantly higher inhibition relative to the selected antivenoms, a demonstration of their superior specificity (% inhibition at highest concentration: 63.28%, 22.83%, and 21.32% for test mAbs, VINS^TM^, and Inoserp^TM^, respectively). Given that the mAbs successfully showed in vitro activity, a demonstration of their ability to abrogate *N. ashei* venom-induced cytotoxicity and perhaps lethality is crucial in testing their potential utility in snakebite therapy. Nevertheless, the results showed a positive step in the production of antivenoms targeted at key venom components that are clinically significant. Future studies involving the development of anti-3FTxs antibodies against some of the species included in the production of both VINS^TM^ and Inoserp^TM^ antivenoms may be important in comparing the activity of such antibodies with the current anti-3FTxs mAbs and the two commercial antivenoms.

## 5. Materials and Methods

### 5.1. Three-Finger Toxins and Venoms

Three-finger toxin fraction was purified using size-exclusion chromatography as described elsewhere [30]. *N. ashei* venom was obtained in its lyophilized form from snakes kept at the Bio-Ken snake farm in Watamu, Kenya. Venom was reconstituted in distilled water and stored at −20 °C prior to use.

### 5.2. Antivenoms

Two commonly used antivenoms, Inoserp^TM^ (Inosan, B. NO: 0IT01002; expiry date: January 2023) and VINS^TM^ (African HIS, B. NO: 07AS18005; expiry date: April 2022) were obtained commercially. Reconstitution was done according to the manufacturer’s instructions.

### 5.3. Animals

Eight-week-old adult female BALB/c mice (*n* = 6) were used for the study. Mice were maintained at the Kenya Medical Research Institute animal facility (KEMRI). Food and water were provided *ad libitum*.

### 5.4. Ethical Review

Ethical approval for the study was obtained from KEMRI Scientific and Ethical Review Unit with protocol number KEMRI/SERU/CBRD/229/4340.

### 5.5. Optimization of ELISA Parameters

ELISA parameters (antigen coating concentration, sample antigen concentration, primary and secondary antibody concentrations, and substrate sensitivity) were optimized as described elsewhere [31].

### 5.6. Generation of Monoclonal Antibodies

Production of hybridomas was carried out using the method described by Himananto and colleagues [32] with modifications. Two groups of two 8-week-old adult female BALB/c mice were each immunized with 50 µg and 100 µg three-finger toxin antigen respectively. Toxin was attenuated in 0.125% glutaraldehyde solution prior to immunization. For both the primary and booster immunizations, a final volume of 200 µL comprising the attenuated toxin and adjuvant (Freund’s Complete Adjuvant and Freund’s Incomplete Adjuvant (Sigma Aldrich, Darmstadt, Germany), respectively) were injected intraperitoneally. Mice were bled by tail puncture at intervals of two weeks and boosters administered immediately for a period of 14 weeks. Antibody screening was done using an indirect ELISA described elsewhere [31]. The mouse with the highest antibody titer was identified, given a booster (50 µg), and sacrificed 72 h later for fusion. Following splenectomy, isolated mouse spleen cells were fused with SP2/0 myeloma cells in a 3:1 ratio in the presence of 50% (*w/v*) polyethylene glycol (PEG) 1500 (Roche, Indianapolis, IN, USA). Successfully fused cells (hybridoma) were selected via culturing using hypoxanthine aminopterin thymidine (HAT) medium constituted using RPMI medium (Nissui Pharmaceuticals, Tokyo, Japan), 10% fetal bovine serum (FBS) (Gibco, Paisley, Scotland, UK), 50× HAT supplement (Gibco, Paisley, Scotland, UK), and 5% (*v/v*) briclone hybridoma cloning additive (Thermo Fisher Scientific, Horsham, UK). Cultures were expanded and screened for antibody production against 3FTx antigen using indirect ELISA previously indicated. Positive cultures that produced anti-3FTxs antibodies were cloned using limiting dilution to generate monoclonal cultures of antibody-producing hybridoma cells.

### 5.7. Screening of Serum and Hybridoma Supernatants for Antibodies

Mice serum samples and hybridoma supernatants were screened for antibodies using an indirect ELISA described elsewhere [31]. Briefly, wells of a 96-well maxisorp plate (NUNC, Roskilde, Sjælland, Denmark) were coated with 1 µg/mL of the toxin prepared in carbonate-bicarbonate buffer, pH 9.6, and incubated at 4 °C overnight. The plate was washed with 200 μL of washing buffer (2% BSA + 0.05% PBS-Tween 20, pH, 7.4), with 3 min between each wash. Two hundred microliters (200 µL) of the blocking buffer (same as washing buffer) was then added to each well and incubated at room temperature for 60 min. The plate was washed again as previously described. One hundred microliters (100 µL) of mice serum samples prepared at 1:1000 in the blocking buffer was added to the wells in duplicates and incubated at 37 °C for 60 min. For hybridoma supernatants, 100 µL was added directly without dilution. The plate was washed and goat anti-mouse HRP-conjugated antibody (American Qualex, San Clemente, CA, USA) diluted 1:1000 in the blocking buffer was added to each well. The plate was incubated at 37 °C for 60 min, washed, and 200 µL of substrate (5 mg O-phenelynediamine-OPD, Sigma Aldrich, St. Louis, MO, USA) was added to each well. The plate was incubated at 37 °C for 60 min in the dark and the reaction stopped by adding 50 µL of stop solution (3M H_2_SO_4_). The plate was analyzed at 492 nm on a plate reader (Multiscan EX reader, Thermo Scientific, Waltham, MA, USA).

### 5.8. Characterization of mAbs

The test mAbs were purified from hybridoma supernatant using the MAbTrap^TM^ Kit column (GE Healthcare Bio-sciences AB, Uppsala, Sweden). Briefly, the kit was set up and connected to a low adaptor. All buffers were prepared in accordance with the manufacturer’s instructions. The column was equilibrated with 5 mL distilled water at a flow rate of 2 drops/sec. Hybridoma supernatants were then applied to the column after which the column was rinsed with 5 mL binding buffer. Antibodies were then eluted by applying 10 mL elusion buffer and 1 mL fractions collected in labeled tubes containing 75 µL neutralizing buffer per tube. Eluted fractions were then analyzed using the Qubit Protein Assay Kit (Life Technologies, Carlsbad, California, USA) to determine the immunoglobulin yield. Immunoglobulin concentration was determined using the Qubit Protein Assay Kit (Life Technologies, Carlsbad, California, USA). The purified mAbs were then isotyped using the IsoStrip mouse monoclonal antibody isotyping kit (Roche Diagnostics, Mannheim, Germany). The two assays above were performed in line with the recommendations of the manufacturers.

#### SDS-PAGE Analysis of mAbs

The purity of the test mAbs (P4G6a, P6D9a and P6D9b) was analyzed on a 15% polyacrylamide gel electrophoresis (SDS-PAGE) in line with the method described by Laemmli and colleagues [33] with modifications. For each mAb, a 100 µL sample was prepared, comprising 70 µL mAbs, 25 µL 4x LDS sample buffer (Van Allen Way, Carlsbad, CA, USA), and 5 µL DTT (Thermo Scientific, Waltham, Massachusetts, USA). Samples were heated on a heat block for 5 min at 90 °C, loaded into the wells and run at 20 mA for 80 min alongside a 10–250 kDa precision plus protein standard (Bio-Rad Laboratories Inc., Hercules, CA, USA). Staining of the protein was done using Coomassie brilliant blue dye for 30 min with gentle shaking after which the gel was de-stained overnight with gentle shaking on a rotator. Gel was then visualized for the presence of bands.

### 5.9. Monoclonal Antibody Titration Using ELISA

The purified test mAbs were evaluated for their binding efficacy to the target antigen employing an ELISA titration test described by Fauches and colleagues [21]. Briefly, 1 µg/mL of purified 3FTx antigen prepared in coating buffer was adsorbed to the ELISA plate overnight at 4 °C. The plate was blocked at room temperature for 60 min and washed three times. Each of the three clones (P4G6a, P6D9a, and P6D9b) were diluted two-fold in blocking buffer and 100 µL loaded in duplicates. The clones were assayed alongside the pre-immune serum sample as negative control. The plate was incubated for 60 min at 37 °C, washed three times, and 100 µL of goat-anti mouse HRP-conjugated antibody (1:1000) was added to the wells. The plate was again incubated for 60 min at 37 °C and washed as previously described. Two hundred microliters (200 µL) of 5 mg O-phenelynediamine substrate was added to the wells, incubated at 37 °C for a further 60 min in the dark, and the reaction stopped with 3M H_2_SO_4_. The plate was then analyzed at 492 nm on a plate reader.

### 5.10. Evaluating Inhibitory Activity of Generated mAbs

The activity of the purified test mAbs was evaluated using an inhibition ELISA model described elsewhere [31]. The activity of the mAbs was premised on the ability of coated 3FTx antigen and crude *N. ashei* venom-containing 3FTxs to competitively bind anti-3FTxs mAbs, thereby inhibiting the coated antigen. Following the coating of the plate (plate A) with 1 µg/mL of purified 3FTx antigen overnight and subsequent washing and blocking, a three-fold serial dilution of crude *N. ashei* venom (27 µg/mL) was performed from row A to H of a 96-well culture plate (plate B). One hundred and fifty microliters (150 µL) of the crude venom (constituted in blocking buffer) was added to duplicate wells in the first rows only (plate B). Fifty microliters (50 µL) was then transferred to the wells in rows B to G containing 100 µL of blocking buffer. The ‘no antigen control’ (NAC) wells (row H) had blocking buffer without the crude venom (sample antigen). Separate primary antibodies comprising of cocktail of test mAbs, VINS^TM^, and Inoserp^TM^ antivenoms as well as mice polyclonal antibodies (3FTx-challenged mice) were diluted two-fold in the blocking buffer and 100 µL was loaded into the respective wells including the ‘NAC’ wells. Cocktail of the test mAbs was prepared by pulling together equal volumes of each mAb. The plate was then incubated at 37 °C for 60 min, after which the complex was transferred to plate A for a further one-hour incubation. The plate was washed three times and 100 µL of goat-anti mouse HRP-conjugated antibody (1:1000) was added to the wells. After incubating the plate for 60 min at 37 °C, it was washed again, and 200 µL of OPD substrate was added to the wells. The plate was further incubated for 1 h, 50 µL of stop solution was added, then read at 492 nm. The % inhibition was then calculated in the various wells using the formula below:

$$\mathrm{Percent}\;\mathrm{inhibition}=\;\frac{\mathrm{NAC}\;\mathrm{OD}-\mathrm{Test}\;\mathrm{sample}}{\mathrm{NAC}\;\mathrm{OD}}\;*\;\;100$$

### 5.11. Statistical Analysis

One-way ANOVA was used to determine differences between groups and then proceeded by Tukey or Dunnette’s multiple comparison tests. For statistical significance, a *p*-value < 0.05 was set for all comparisons.

## 6. Patents

A patent application was filed with the Directorate of Intellectual Property Management and University-Industry Liaison of the Jomo Kenyatta University of Agriculture and Technology, Kenya.

## Data Availability

The data presented in this study are available in this article.

## Sample Availability

Samples of the antibodies are available from the authors.
